# Coagulation and Fibrinolysis in Kidney Graft Rejection

**DOI:** 10.3389/fimmu.2020.01807

**Published:** 2020-08-25

**Authors:** Giovanni Stallone, Paola Pontrelli, Federica Rascio, Giuseppe Castellano, Loreto Gesualdo, Giuseppe Grandaliano

**Affiliations:** ^1^Nephrology, Dialysis and Transplantation Unit, Department of Medical and Surgical Sciences, University of Foggia, Foggia, Italy; ^2^Nephrology, Dialysis and Transplantation Unit, Department of Emergency and Organ Transplantation, University of Bari Aldo Moro, Bari, Italy; ^3^Nephrology Unit, Department of Translational Medicine and Surgery, Università Cattolica del Sacro Cuore, Rome, Italy; ^4^Department of Medical and Surgical Sciences, Fondazione Policlinico Universitario “A. Gemelli” IRCCS, Rome, Italy

**Keywords:** coagulation, fibrinolysis, protease-activated receptors, innate immunity, graft function

## Abstract

Coagulation system is currently considered an integrated part of innate immunity. Clotting activation in response to bacterial surface along with complement cascade priming represents the first line of defense against pathogens. In the last three decades, we learned that several coagulation factors, including factor II or thrombin and factor X, can interact with specific cell surface receptors activated by an unusual proteolytic mechanism and belonging to a novel class of G-protein-coupled receptors known as protease-activated receptors (PARs). PARs are expressed by a variety of cells, including monocytes, dendritic cells, and endothelial cells and may play a key role in the modulation of innate immunity and in the regulation of its interaction with the adaptive branch of the immune system. Also, the fibrinolytic system, in which activation is controlled by coagulation, can interact with innate immunity, and it is a key modulator of extracellular matrix deposition eventually leading to scarring and fibrosis. In the setting of kidney transplantation, coagulation and fibrinolytic systems have been shown to play key roles in the ischemia/reperfusion injury featuring delayed graft function and in the pathogenesis of tissue damage following acute and chronic rejection. In the present review, we aim to describe the mechanisms leading to coagulation and fibrinolysis activation in this setting and their interaction with the priming of the innate immune response and their role in kidney graft rejection.

## Introduction

Kidney transplantation is the treatment of choice for most patients with end-stage renal disease because kidney graft recipients live longer than dialysis patients and have a markedly higher quality of life. The improvement in the immunosuppressive drugs and protocols dramatically reduced the clinical impact of acute rejection and significantly enhanced graft survival. However, graft half-life is still far from being ideal. The quality of the grafts is significantly worsening, and they are more exposed to damage in immediately before and after transplantation, as a consequence of brain death and cold/warm ischemia (1). These peri-transplant events are often mediated by innate immunity and, therefore, are poorly influenced by current immunosuppressive approaches (2). In addition, we are now realizing that they are significantly associated with long-term transplant outcome and with the occurrence of rejection. It is conceivable, then, that the priming of innate immunity early posttransplantation may significantly amplify the adaptive response to the graft (3, 4). Thus, the need to improve our therapeutic approaches to limit the effects of the innate arm of host immune system is now clear. The innate response has cellular and soluble components. Among the latter, complement cascade received in the last decade a considerable attention, and several drugs able to influence the activation of this proteolytic system have been introduced in the clinical scenario (5). However, complement is not the only enzymatic cascade activated in the innate immune response. Indeed, there is an increasing body of evidence that also the coagulation system may play a key role in this setting.

## Coagulation Cascade and Innate Immunity

The coagulation system is a proteolytic cascade that plays a key role in blood clotting. In the last decades, several intersection points between coagulation and immunity have been delineated, and the coagulation system is currently considered an integrated part of innate immune immunity.

**Figure 1:**
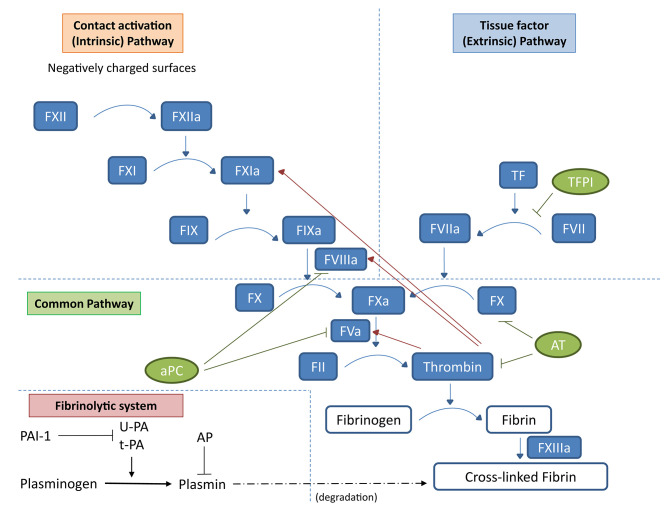
Schematic representation of the coagulation cascade and the fibrinolytic system. In the coagulation cascade, the *extrinsic pathway* starts with tissue factor (TF) and activated factor VII (FVIIa). Tissue factor in physiological conditions is not in contact with the blood; however, damage of blood vessel structure can expose this membrane-associated glycoprotein to the blood, thus activating the extrinsic pathway of the coagulation cascade. TF can also be expressed by mononuclear cells or endothelial cells in response to inflammatory mediators. The TF/FVIIa complex promotes the activation of factor X (FXa). In the *intrinsic pathway*, negatively charged surfaces (such as phospholipids and polyphosphates from activated platelets) activate FXII, initiating a cascade leading to FXa. The *common pathway* FXa, along with the cofactor FVa, converts prothrombin (FII) to thrombin (FIIa). Finally, thrombin activates fibrinogen into fibrin and FXIIIa acts on fibrin strands to form a fibrin mesh. Regulation of coagulation activation occurs by three distinct natural anticoagulant pathways: AT (which blocks FXa and thrombin), TFPI (which inhibits the tissue factor–factor VIIa complex), and aPC (which proteolytically degrades factor Va and factor VIIIa). Natural regulators of the coagulation cascade are indicated in green: TFPI, AT, and aPC. Once the first thrombin is produced, it induces the propagation of the coagulation cascade (red arrows) but also a feedback regulation through aPC. In the fibrinolytic system, uPA and tPA catalyze the proteolysis of plasminogen into plasmin, which, in turn, degrades fibrin. Inhibition of the plasminogen system occurs by specific PAIs and by α2-AP at the level of plasmin.

Traditionally, the coagulation system has been classified into extrinsic and intrinsic pathways. These two pathways converge, forming the common pathway, which results in the activation of factor X to Xa. In the extrinsic pathway, tissue factor exposed by vascular injury interacts with plasma factor VIIa and activates factor IX and X, with results in the formation of small amounts of the serine protease thrombin. Thrombin not only acts on fibrinogen to form the fibrin clot but also stimulates platelet and vascular endothelial cells and activates plasma coagulation factors, priming the intrinsic pathway (6). Blood-circulating FXII is autoactivated by contact with negatively charged surfaces, such as nucleic acids, platelet, and microbial polyphosphate and thus converted to the serine protease FXIIa (7) finally promoting the sequential activation of coagulation factors XI and IX (Figure 1). Regulation of coagulation activation occurs by three distinct physiological anticoagulant pathways: antithrombin (AT) (which blocks factor Xa and thrombin), tissue factor pathway inhibitor (TFPI) (which inhibits the tissue factor–factor VIIa complex), and activated protein C (aPC) (which proteolytically degrades factor Va and factor VIIIa).

The coagulation cascade can be directly activated by different pathogens, including bacteria and viruses (8–12), and the formation of fibrin clots can trap bacteria and limit the invasiveness of pathogens. However, several other pathological conditions are characterized by the activation of the coagulation system, and studies performed in the past 15 years have provided novel insights into the role of coagulation proteases in kidney disease beyond their function in normal hemostasis and thrombosis (13).

## Cross Talk Between Coagulation and Complement System

Activation of the coagulation proteolytic cascade can influence innate immunity in different ways by interacting with diverse molecular mechanisms involved in the immune response. One of the main columns of innate immunity is represented by the complement system, and several possible interactions between the two cascades have been proposed (14).

The complement system is an important component of the innate immunity and functions primarily as a first-line host defense against pathogenetic infections and in the removal of immune complexes and apoptotic cells (15). The complement system can be activated by three main pathways—classical, lectin, and alternative—that include several components and regulators, produced by different cells under diverse conditions finally leading to a proteolytic cascade, which terminate in opsonization and lysis of pathogens as well as in the generation of proinflammatory molecules (16). The classical pathway uses C1 and is triggered by antigen–antibody immune complexes. It consists in the activation of the serine proteases C1r and C1s, the subsequent cleavage of C4 and C2, and the generation of the classic C3 convertase (C4bC2a), which cleaves C3 into the anaphylatoxin C3a and C3b. The lectin pathway activation also leads to the formation of C4bC2a C3 convertase complex but is activated by opsonin, mannose-binding lectin (MBL), and ficolins, instead of C1q. Finally, the alternative pathway is constitutively active at low levels in the normal host as a result of spontaneous C3 hydrolysis. It is regulated by factor H and factor I and need factor B and factor D to generate the alternative pathway C3 convertase (17). The terminal phase is similar for the classical, lectin, and alternative pathways. The incorporation of C3b in the C3 convertases results in the formation of the C5 convertases that cleave C5 into C5a and C5b, leading to the formation of the multimeric terminal membrane attack complex (C5b-9) (18).

Other factors of the coagulation and fibrinolytic pathway including thrombin; human factors XIa, Xa, and IXa; and plasmin can cleave C5 without the involvement of other complement factors, leading to the so-called extrinsic complement pathway (19).

Proteases of the lectin pathway induce thrombin and fibrin generation, stabilize the fibrin clot, and impair fibrinolysis (20). Takahashi K et al. demonstrated that the MBL and MBL-associated serine protease (MASP)-1/3 together can function as thrombin (21). In particular, they observed both *in vitro* and *in vivo* in MBL null mice that MBL deficiency may lead to disseminated intravascular coagulation and organ failure during infectious diseases (21). MASP-1 is a serine protease able to cleave several proteins, both complement and non-complement substrates, in the human blood. MASP-1 shares many characteristics with thrombin; for example, it can cleave several members of protease-activated receptors (PARs)—PAR-1, PAR-2, and PAR-4—thus leading to cytokine production leading to chemotaxis of neutrophils (22). MASP-1 is also involved in coagulation and thrombus formation by the activation of endothelial cells and generation of thrombin (22).

Several studies also reported that the coagulation system can activate the lectin pathway in turn (23, 24). These interactions can play an important role in clinical conditions because they can influence hypercoagulability and increase thrombosis risk.

Coagulation factor II or pro-thrombin, once activated into thrombin, can prime the complement system through a direct interaction with C5 (25). On the other hand, it is now clear that complement activation through C5 splits products and that C5b-9 can trigger the coagulation cascade promoting tissue factor expression by several cell types, including monocytes and endothelial cells (26). This positive feedback loop represents a powerful amplification mechanism of innate immune system activation.

Tissue factor is emerging as an important player not only in hemostasis and thrombo-inflammatory diseases but also in non-coagulant signaling pathways mainly through the family of G-protein-coupled receptors PARs (27).

Thrombin, other than having a key role in coagulation cascade, modulates immune and non-immune cell functions interacting with PARs (28). These seven transmembrane domains proteins are activated by a peculiar proteolytic mechanism. The protease cleaves the N terminal extracellular domain of the receptor, leading to the exposure of a tethered ligand that interact with the extracellular loop 2 domain and initiate receptor signaling. Thrombin can activate PAR-1, PAR-3, and PAR-4 (29). Interestingly, thrombin is not the only coagulation factor that can interact with PARs. Indeed, coagulation factors VIIa and Xa are known activators of PAR-2 (28). PARs are expressed by several immune cells, thus representing the main link between coagulation and innate immunity (30), and their expression levels have been described as implicated in the pathogenesis of several kidney diseases (31).

Through their activation, coagulation factors may play a central role in promoting the release of pro-inflammatory cytokines and chemokines from different cell types including dendritic cells, monocytes, lymphocytes (32–34), endothelial cells (35, 36), glomerular mesangial cells, pericytes, and epithelial cells (37) participating in the modulation of the innate and adaptive arms of immune response. It has been recently demonstrated that interleukin (IL)-1α is directly activated by thrombin pointing the importance of this pathway not only for normal physiology but also in the pathogenesis of inflammatory and thrombotic diseases (38). Similarly, several factors in the coagulation and fibrinolytic pathways can trigger either proinflammatory or anti-inflammatory host responses.

Coagulation and intravascular thrombus growth *in vivo* can be induced by neutrophil-derived serine proteases and nucleosomes (39). Neutrophil serine proteases play an important role as regulators of cell signaling and immune response, in particular against microbial threats (40). Interestingly, neutrophils produce a pool of FXII, functionally distinct from hepatic-derived FXII, specifically involved into neutrophil trafficking at sites of inflammation, through urokinase plasminogen activator (uPA) receptor (uPAR) and Akt signaling (41). FXIIa-induced neutrophil activation is also involved in macrophage polarization and induces T-cell differentiation, all contributing to host defense against pathogens (42).

## Coagulation Cascade and Ischemia/Reperfusion Injury

The coagulation system activation has been suggested to play a significant role in several pathologic processes involving the kidney graft such as the early posttransplant period (43). In this first phase, the main issue is represented by the exposure of the graft to ischemia and a subsequent reperfusion. It is well known that ischemia/reperfusion injury is a leading cause of acute kidney injury (AKI) (44). In the setting of kidney transplantation, the graft experiences a prolonged cold ischemia after harvesting and brief warm ischemia followed by reperfusion during the transplantation procedure. The tissue damage featuring ischemia/reperfusion in renal transplant recipient is represented by tubular cell apoptosis and interstitial inflammation, a pathogenic event underlying an early posttransplant form of AKI known as delayed graft function (DGF) (45). This early posttransplant event, complicating between 15 and 30% of kidney transplantations, does not only represent a delay in graft function recovery but dramatically influences the long-term outcome of the transplanted organs. Indeed, there are several evidences that grafts experiencing DGF present a significantly lower survival (46); thus, prevention of DGF might significantly improve the long-term outcome of kidney grafts.

The key role of innate immunity in transplant-associated ischemia/reperfusion damage leading to DGF is clearly suggested by the strong expression of pro-inflammatory mediators, cytokines, and chemokines and by the priming of the coagulation cascade leading to monocyte–macrophage recruitment and interstitial infiltration (47–49). The coagulation cascade is strongly activated at the time of transplantation mainly owing to the induced vascular expression of tissue factor after ischemia/reperfusion (50). Damage of blood vessels induces the formation of tissue factor-activated factor VII, which, in turn, activates factor X and ultimately activates factor II (prothrombin) to IIa (thrombin).

There are several direct and indirect evidences that suggest the activation of coagulation at this stage as a key potential mediator in determining graft quality and outcome (37, 51).

Thrombin, locally released, may contribute to inflammation by the stimulation of PARs expressed by several resident cells including endothelial, tubular epithelial, and mesangial cells with the subsequent activation of signaling pathways leading to the production of cytokines and growth factors (52). Akt-mammalian target of rapamycin-S6k and NF-kB-inducing kinase (NIK)-NF-kB axis are two signaling pathways regulating cell survival and inflammation. The activation of both Akt and NIK-NF-kB signaling was detected in graft biopsies from DGF patients and were both triggered by thrombin in cultured proximal tubular cells, inducing a nuclear translocation of the active form of Akt and a time-dependent increase of NIK phosphorylation (53). Activated Akt is able to phosphorylate several substrates in the cytoplasm and nucleus. In transplantation, Akt-mTOR-p70S6k pathway is inhibited by rapamycin. Indeed, the use of this treatment is associated with prolonged period of DGF (54). On the other hand, NF-kB induces several genes involved in inflammatory response (55). In addition, the simultaneous activation of these two keys signaling pathways in a pig model of ischemia/reperfusion injury and in human biopsies from DGF correlated with tubulointerstitial and glomerular fibrin deposition in both cases (53). Besides participating in the activation of innate immunity featuring ischemia/reperfusion injury underlying DGF, the activation of the coagulation system in this setting might represent a pivotal element in the priming of the adaptive alloimmune response. Indeed, infiltrating dendritic cells during DGF express PAR-1 and are localized in the proximity of fibrin and C3 deposits (56). *In vitro* PAR-1 activation on dendritic cells caused a significant upregulation of C3 and complement receptor expression. In addition, thrombin caused a marked reduction of IL-10 mRNA abundance and an increase of IL-12/IL-17 p40 gene expression and promoted C3a ability to increase IL-12/IL17 mRNA abundance. These changes can influence dendritic cells ability to induce interferon-γ production by T cells, thus suggesting the activation of a T helper-1 bias (56).

Another factor associated with renal damage after kidney transplantation is increased tissue factor expression. Tissue factor is one of the main activators of the coagulation system; it is a membrane component of many cells and is released in blood plasma after a cell damage. It has been recently demonstrated that recipients with postoperative complications had much higher concentrations of tissue factor in renal vein in the first minute during reperfusion, which may be associated with kidney damage (57).

Altogether, these data strongly support the strict correlation between the signaling pathways activated by coagulation factors in resident and infiltrating cells and the ischemia/reperfusion damage. Thus, the coagulation cascade might represent a potential therapeutic target to reduce ischemia/reperfusion-induced injury in the attempt to lessen the incidence of DGF.

## Coagulation Cascade and Chronic Rejection

Interstitial activation of the coagulation cascade is also a common feature of tubular-interstitial nephritis and in particular of renal graft rejection. Chronic allograft rejection is the final common pathway of progressive graft injury and is characterized by interstitial fibrosis and tubular atrophy along with a variable degree of chronic glomerular changes, and this condition is associated with both glomerular and interstitial fibrin deposition (58). Fibrin is known to induce migration and proliferation of the major cell types involved in interstitial fibrosis, such as macrophages and vascular smooth muscle cells (59, 60). In addition, PAR-1 expression is significantly increased at both the glomerular and tubular levels in chronic graft rejection, clearly suggesting that thrombin may significantly modulate resident cell activation in this setting (58).

Following kidney transplantation, microvascular thrombi along with endothelial dysfunction and fibrin deposition in the kidney graft were recognized as a challenging cause of graft dysfunction, particularly for highly sensitized kidney transplant recipients. Indeed, in these patients, the activation of endothelial cells by the involvement of both complement and coagulation pathways is a response to human leukocyte antigen (HLA) antibodies (61).

*In vitro* study suggests that incubation with specific anti-HLA antibody against a line of human umbilical endothelial cells induces upregulation of tissue factor expression and activity, defining a role in direct initiation of coagulation by HLA antibody binding (62). Yamakuchi et al. have shown that, both *in vitro* and *in vivo*, anti-HLA antibody induces endothelial cell exocytosis and, in turn, an increased expression of von Willebrand factor (vWF) (63).

Increasing evidence underlie that in response to HLA antibodies, both complement and coagulation cascades can be activated (20, 61); however, traditional views of HLA antibodies have focused on the initiation of complement, and studies relating to the initiation of coagulation by HLA antibody activation are still limited. Understanding the complexities of these interactions remains a challenge especially within the scope of intervention.

## The Fibrinolytic System in Innate Immunity

The fibrinolytic system removes fibrin from the vascular system, preventing clots from occluding the vessel. The activation of the fibrinolytic system is controlled by coagulation itself. Activation of the fibrinolytic system depends mainly upon uPA and tissue-type plasminogen activator (tPA) that catalyze the proteolysis of plasminogen into plasmin, which, in turn, degrades fibrin, preventing its extracellular deposition (Figure 1). uPA and tPA, with the glycosylphosphatidylinositol (GPI)-linked uPAR, are expressed by a variety of cells of hematopoietic origin and are upregulated during infections and inflammation (64). The interaction between the plasminogen and complement systems at sites of tissue injury represents an important bridge between innate and adaptive immunity (65). Indeed, the activating effect of plasmin on complement cascade both *in vitro* and *in vivo* is well known (66).

The plasminogen system is also important for tissue remodeling, in particular through its specific endogenous inhibitor plasminogen activator inhibitor-type 1, PAI-1. Inhibition of the plasminogen system occurs by specific PAIs and by α2-antiplasmin (AP) at the level of plasmin (Figure 1). PAI-1 is not expressed in the healthy kidney (67). In contrast, its expression is significantly increased in several primary and secondary glomerulonephritis, and several evidences suggest a key role for this protease inhibitor in the progression of renal damage in this setting (68). In particular, PAI-1 may play an inhibitor effect also on different proteases involved in the degradation of extracellular matrix, including metalloproteases, thus promoting tissue fibrosis.

## Fibrinolysis Activation in Kidney Transplantation

The key role of fibrinolytic system is to dissolve blood clots. Plasmin is converted in the active form from plasminogen by the action of tPA. Fibrinolysis deficiency often occurs not only in hemodialyzed patients (69) but also in kidney transplant recipients. The association between fibrinolytic decrease and impaired renal function would be supported by the observation that successful renal transplantation is characterized by an improved fibrinolysis (70), although transplant patients continue to present hypofibrinolysis, which, however, unlike in dialysis patients, is secondary to a rise in PAI-1 (71). A role in the increase of the plasma levels of PAI-I in transplant patients is, indeed, played by the immunosuppressive therapy and in particular by corticosteroids and cyclosporine, in addition to the effects of metabolic disorders featuring transplant recipients, such as insulin resistance or dyslipoproteinemia (71).

Fibrinolysis activation can be influenced by the cause of cadaveric kidney donors' death. Zietek et al. demonstrated that organ donors who had injured death, such as road traffic injury, were characterized by an intensive activation of fibrinolytic process when compared with non-injured donors, which showed instead intensive activation of blood coagulation (72). This observation suggests that injured donors have a lower risk of microthrombi and fibrin deposits than non-injured donors; however, the clinical importance of this observation and the effect on the outcome of the graft are still unknown.

Renal ischemia/reperfusion injury leading to DGF is characterized by an increased expression of uPA and uPAR (73). uPAR plasma concentrations in kidney allograft recipients have been demonstrated to be significantly and negatively correlated with graft function in deceased-donor renal transplant recipients (74). uPAR plays an important role in the structure and function of the renal filtration barrier (75); thus, its upregulation is responsible of proteinuria (72). It has also been demonstrated that tPA regulates renal neutrophil influx in kidney ischemia and reperfusion injury (76), and recent evidences propose a proinflammatory role for postischemic tPA release (77). However, further and larger clinical investigations are needed.

Glomerular and interstitial fibrin deposition observed in chronic renal allograft failure is caused by, other than the pro-fibrotic effect of thrombin, a dysregulation of the plasminogen/plasmin system that results in a subsequent inhibition of fibrinolysis (78). Corticosteroids may induce PAI-1 expression in tubular cells *in vitro* and *in vivo* in chronic renal graft failure (58). Thrombin itself can induce at the tubular and endothelial levels PAI-1 gene and protein expression (13). Immunosuppressive therapy can significantly influence these events. Indeed, rapamycin, an mTOR inhibitor, has a beneficial effect on chronic graft injury progression because patients converted from calcineurin inhibitors to rapamycin present a significant reduction in the progression of interstitial fibrosis and glomerulosclerosis (79). Interestingly, in these patients, the introduction of rapamycin significantly reduced glomerular and tubulointerstitial expression of PAI-1. *In vitro* data demonstrate that rapamycin reduced PAI-1 expression induced by both thrombin and CD40L in proximal tubular cells (79).

## Targeting Coagulation Factors to Limit the Activation of Innate Immunity

A potential limit in the use of activated coagulation factors as therapeutic targets to limit the activation of the innate immunity is represented by their persistence within the fibrin clots where they are usually protected from the majority of their soluble natural or synthetic inhibitors. The fibrin deposition due to the activation of the coagulation system is in fact under the tight control of fibrinolysis, a highly regulated enzymatic process that prevents accumulation of intravascular fibrin (80).

Several animal models have been used to demonstrate the specific effect of coagulation inhibition on the graft outcome. In a porcine preclinical model of renal auto-transplantation, peri-transplantation treatment with an anti-factor Xa compound protected kidney grafts, improving functional recovery and reducing chronic lesions (81). The same authors demonstrated in this animal model the benefits of a preservation anticoagulation therapy using a specific and effective dual-molecule anti-coagulation factors Xa/IIa, which was able to protect the kidney by reducing thrombin generation with subsequent early functional recovery and decreased chronic lesions (82). These results opened the way to the potential therapeutic benefits of the use of anticoagulation in the reperfusion solutions, an approach that may significantly limit the potential side effects due to inhibition of the coagulation system in a surgical setting. Although phase I human studies have been conducted on these synthetic anticoagulants with a dual mechanism of action (83), their use in the clinical practice needs to be further investigated. One approach to possibly improve outcomes after transplantation is the use of C1 inhibitor, a serum protease inhibitor (serpin) that binds covalently and inactivates C1r, C1s, and mannan-binding protein blocks. Other than the classical and lectin pathways of complement activation, C1 inhibitor also inhibits the contact, coagulation, and kinin systems (84). It has been demonstrated in some trials and studies in kidney transplant recipients that C1 inhibitor treatment may reduce ischemia/reperfusion injury and DGF (85); however, small patient numbers are included in these studies, most results are from single centers, and differences in study design preclude meta-analyses.

## Concluding Remarks

There is an increasing body of evidence that suggests that the activation of the coagulation system is associated with several pathological conditions of the graft and that it is invariably associated with a significant activation of the innate branch of the immune system also through a complex interaction with the complement proteolytic cascade. This activation plays a key part in the pathogenesis of the progression of graft injuries independently of their etiology. It is conceivable that in this setting coagulation cascade activation might mediate the activation of the innate immune system and might be involved, through its effects on dendritic cells, in the amplification of alloantigen-specific adaptive response. Thus, coagulation might be considered a potential therapeutic target to modulate innate immunity and to prevent progressive graft damage. Ideally, the inhibition of coagulation activation or of its downstream cellular effects should be associated with the inhibition of the harmful effects of the complement cascade priming. Targeting the common checkpoints shared by the two proteolytic systems might represent a promising therapeutic approach to reduce the activation of the innate immune system in kidney transplantation and reduce its deleterious consequences on graft function and survival.

## Author Contributions

GS, PP, LG, and GG designed, wrote, and critically revised the review. FR analyzed fibrinolysis activation in kidney transplantation. GC analyzed the cross talk between coagulation, fibrinolysis, and complement system. All authors contributed to the article and approved the submitted version.

## Conflict of Interest

The authors declare that the research was conducted in the absence of any commercial or financial relationships that could be construed as a potential conflict of interest.
